# 267 Impact of a Single-Set Blood Culture Restriction on the Epidemiology of Bloodstream Infections During the 2024 National Bottle Shortage

**DOI:** 10.1017/ash.2026.10448

**Published:** 2026-06-23

**Authors:** Samuel Cincotta, Courtney Victor, Caleb Cox, Mohammed Khan, Dumbani Kayira, Paige Gable, Heather Moulton-Meissner, Richard Stanton, Cayman Doran, Bethany Lavender, Kristen Clancy, Thomas Ewing, Marissa Howard, Alyssa Kent, Maroya Walters, Trent Gulley

**Affiliations:** 1 CDC; 2 Centers for Disease Control and Prevention; 3 Indiana Department of Health; 4 Centers for Disease Control; 5 Orise

## Abstract

**Background:** Extended-spectrum beta-lactamase (ESBL)-producing Enterobacterales are antimicrobial resistant pathogens which cause difficult-to-treat infections. In April 2025, Hospital A notified the Indiana Department of Health about an outbreak of ESBL-Klebsiella pneumoniae. CDC assisted with identifying the sources of the outbreak and recommending control measures. **Methods:** We defined confirmed cases as ESBL-producing K. pneumoniae multi-locus sequence type (ST) 661 (or closely related ST) by whole genome sequencing (WGS). Suspect cases included 3rd- and 4th-generation cephalosporin-resistant K. pneumoniae without WGS data. All isolates originated from patient cultures collected on or after January 1, 2025, from Hospital A’s network of hospitals and outpatient clinics. An incident case was the first case identified from a patient. Among confirmed incident cases, we abstracted medical charts for 30 randomly selected patients. We assessed infection prevention and control (IPC) practices and sampled the hospital environment to identify potential reservoirs. **Results:** We identified 70 confirmed and 52 suspect incident cases between January and September. Median patient age was 70 years (range: 19–91 years); 67 (55%) were female. Within 90 days before incident case collection, 28 of the patients with charts abstracted (93%) received care at Hospital A; 27 (90%) received radiologic imaging, mostly CT scans (17/30; 57%) and transthoracic echocardiograms (16/30; 53%). We observed gaps in hand hygiene, personal protective equipment usage, and environmental cleaning. ESBL-K. pneumoniae was recovered from 23 environmental samples, including radiology equipment. WGS revealed that these isolates clustered with patient isolates, showing 0-26 pairwise high-quality single nucleotide variants across a 95.73% core genome. Following the implementation of IPC recommendations, the average number of monthly cases was 21% lower compared to the 9 months prior. **Conclusions:** Our investigation linked the outbreak to gaps in IPC practices and contamination of the healthcare environment, highlighting the often-overlooked role of the environment in the spread of ESBL-producing pathogens.

